# The effect of inhibiting glycinamide ribonucleotide formyl transferase on the development of neural tube in mice

**DOI:** 10.1186/s12986-016-0114-x

**Published:** 2016-08-23

**Authors:** Lin Xu, Li Wang, JianHua Wang, ZhiQiang Zhu, Ge Chang, Ying Guo, XinLi Tian, Bo Niu

**Affiliations:** 1 Department of Biotechnology, Beijing Municipal Key Laboratory of Child Development and Nutriomics, Capital Institute of Pediatrics, Beijing, 100020 People’s Republic of China; 2 Department of Biochemistry and Molecular Biology, Shanxi Medical University, Taiyuan, 030001 People’s Republic of China; 3 Department of Cardiovascular Disease, Chinese PLA General Hospital of Beijing Military Region, Beijing, 100020 People’s Republic of China

**Keywords:** Neural tube defects, Lometrexol, Mice, Metabolism, Proliferation, Apoptosis

## Abstract

**Background:**

Folate deficiency is closely related to the development of neural tube defects (NTDs). However, the exact mechanism is not completely understood. This study aims to induce murine NTDs by inhibiting one of the folate metabolic pathways, *de novo* purine synthesis and preliminarily investigate the potential mechanisms. The key enzyme, glycinamide ribonucleotide formyl transferase (GARFT) was inhibited by a specific inhibitor, lometrexol (DDATHF) in the pregnant mice.

**Methods:**

Pregnant mice were intraperitoneally injected with various doses of DDATHF on gestational day 7.5 and embryos were examined for the presence of NTDs on gestational day 11.5. GARFT activity and levels of ATP, GTP, dATP and dGTP were detected in embryonic brain tissue. Proliferation and apoptosis was analyzed by real-time quantitative polymerase chain reaction (RT-qPCR), immunohistochemical assay and western blotting.

**Results:**

40 mg kg^−1^ body weight (b/w) of DDATHF caused the highest incidence of NTDs (30.8 %) and therefore was selected as the optimal dose to establish murine NTDs. The GARFT activity and levels of ATP, GTP, dATP and dGTP in embryonic brain tissue were significantly decreased after DDATHF treatment. Furthermore, Levels of proliferation-related genes (*Pcna*, *Foxg1* and *Ptch1*) were downregulated and apoptosis-related genes (*Bax*, *Casp8* and *Casp9*) were upregulated. Expression of phosphohistone H3 was significantly decreased while expression of cleaved caspase-3 was greatly increased.

**Conclusions:**

Results indicate that DDATHF induced murine NTDs by disturbing purine metabolism and further led to abnormal proliferation and apoptosis.

**Electronic supplementary material:**

The online version of this article (doi:10.1186/s12986-016-0114-x) contains supplementary material, which is available to authorized users.

## Background

Neural tube defects (NTDs) are common, severe congenital malformations due to the failure of neural tube closure during embryonic development [1, 2]. Studies have shown that folate deficiency is closely related to NTDs and that up to 50 to 70 % of NTDs can be prevented by supplementation of folic acid [3–5]. However, the mechanisms on how folate deficiency causes NTDs still remained unclear. Besides, Heid et al. [6] controlled the folate intake and showed that mouse embryos do not develop NTDs. This indicates that folate deficiency alone is not a cause of NTDs although it is certainly a risk factor in humans and mice. In our previous study, murine NTDs have been successfully induced by inhibiting the key enzyme, DHFR, in folate metabolic pathway [7]. We suppose that folate deficiency refers to folate and folate related metabolic disorder, which may lead to NTDs. Obviously, the inhibition of DHFR results in dysmetabolism of THF and thus one carbon units. Decreased one carbon units will affect biosynthesis of nucleotide and the process of methylation, which may further impact several in vivo physiological processes, including nucleic acid metabolism, cellular proliferation and apoptosis. Defects in any of these processes may lead to the occurrence of NTDs. Glycinamide ribonucleotide formyl transferase (GARFT) is a key enzyme in the process of de novo purine biosynthesis. It converts glycinamide ribonucleotide (GAR) to glycinamide ribonucleotide formyl-acid (FGAR) by using N-10-formyl-tetrahydrofolate as the formyl donor [8]. Therefore, the inhibition of this enzyme may impair the biosynthesis of purine nucleotide [9].

Lometrexol (dideazatetrahydrofolate, DDATHF) can inhibit the activity of GARFT by tightly binding with it and further inhibit de novo purine synthesis, causing decreased single purine nucleotide pool in cells, abnormal cell proliferation and apoptosis, even cell cycle arrest [10, 11]. Reports have shown that the occurrence of NTDs is associated with imbalance between cell proliferation and apoptosis. Studies have proved that disordered proliferation and apoptosis in animal models of NTDs were induced by hyperthermia [12], hyperglycemia [13], cyclophosphamide [14], or methotrexate (MTX) [15]. Therefore, cell proliferation and apoptosis have to be kept in dynamic balance during the formation of neural tube. In the present study, we aim to establish murine model of NTDs by using DDATHF, and investigate the role of abnormal proliferation and apoptosis in the development of NTDs.

At present, NTDs induced by impairment of purine nucleotide synthesis or inhibition of GARFT have not been reported. This study aims to investigate whether the murine model of NTDs can be induced by impairment of purine biosynthesis via inhibition of GARFT using a specific inhibitor, DDATHF, and to further explore the potential mechanisms.

## Methods

### Materials

Chemicals and biochemical were purchased from Sigma-Aldrich (Poole, Dorset, U.K.) unless otherwise indicated.

### Establishment of murine NTDs by using DDATHF

All experimental procedures were reviewed and approved by the Animal Ethics Committee of the Capital Institute of Pediatrics. C57BL/6 mice (7–8 week, 18–20 g, Vital River Laboratory, Beijing, China) were used in the experiment. Mice were housed individually under controlled conditions (22 °C, relative humidity 40 ~ 60 %, 12 h light/dark cycle)-and with free access to food and water. Female mice were mated with males overnight and vaginal plugs were examined in the following morning. The presence of vaginal plug in the pregnant mice was considered as gestational day 0.5. Pregnant mice were randomly divided into seven groups. Six groups were treated with DDATHF (Sigma-Aldrich, St. Louis, MO, USA) by intraperitoneal injection on gestation day 7.5 [15, 30, 35, 40, 45 and 60 mg kg-1 body weight (b/w)]. The control group was intraperitoneally injected with 0.9 % NaCl at the same volume.

### Examination of morphological and pathological changes of embryos

Pregnant mice were sacrificed on gestation day 11.5. All embryos were carefully dissected and examined under a dissect microscope. The brain tissue in control group from one litter (3–4 normal embryonic brain tissues) was treated as a control sample, and that of NTD embryos from one litter (3–4 NTD embryonic brain tissues) was gathered as a NTD sample. All samples were stored at −70 °C. The whole embryonic tissues were Paraffin-embedded and cut into 5 μm thick slices according to the procedures. The slices were then stained with hematoxylin and eosin (H & E) and observed under light microscope.

### Detection of GARFT activities of embryonic tissue

The activity of GARFT was detected according to the previous description with modifications [16]. Briefly, embryos from sex litters (3–4 embryonic tissues per litter) were collected respectively at 0, 6, 24, 48 and 96 h after i.p. injecting of DDATHF (40 mg/kg body weight) at the optimal dose. NTD embryonic brain tissues from one litter were pooled as one sample for analysis. GARFT protein of embryonic brain tissue was extracted using the mammalian tissue lysis/extraction reagent (Sigma-Aldrich). The concentrations of protein were determined by Nanodrop 2000 (Thermo Scientific, Waltham, MA, USA). GARFT activity was determined spectrophotometrically at 298 nm. The extraction of embryonic brain tissue (20 μg) wereincubated with 45 μmol of Tris-HCl (pH 7.5), 90 μmol of 2-mercaptoethanol, 0.20 μmol of α, β-GAR and 40 nmol of 10-formyltetrahydrofolate in 0.9 ml for 1 min at 30 °C. Routine assays were performed with 11 μM 10-formyl-5, 8-dideazafolic acid and 10 μM GAR. One unit of enzyme activity represented the formation of 1 μmol product per minute.

### Measurement of levels of ATP, GTP, dATP and dGTP by HPLC

The content of ATP, GTP, dATP and dGTP in normal and NTD embryonic brain tissue on gestation day 11.5 (3–4 embryos per litter as one sample) was measured by high performance liquid chromatography (HPLC) as described previously [17]. In brief, control and NTD embryonic brain tissue were collected and placed on ice and rapidly frozen in liquid nitrogen. The grinding frozen tissue was mixed and incubated with ice-cold trichloroacetic acid for 20–30 min, then centrifuged at 3000 × g (10 min, 4 °C). The supernatant was neutralized with trichloroacetic acid in cold Freon. A portion of the supernatant was used for HPLC detecting. Agilent Polaris C18-a column was used for the detection (4.6 × 150 mm, 5 μm). The mobile phase was as follows: A: H2O, 10 mM tetrabutyl ammonium hydroxide (TBAH), 10 mM KH2PO4 (pH = 7); B: 10 mM TBAH, of methanol (pH = 7). Gradient elution procedure was as follows: 0–30 min, 40 % B-60 % B; 30.1–60 min, 60 % B. Flow rate: 1.0 ml/min, detection wavelength: 254 nm; injection volume: 50 μl, temperature of column: 25 °C.

### Real-time quantitative polymerase chain reaction (RT-qPCR)

Total RNA from three control and three NTD embryonic brain tissue on gestation day 11.5 were extracted by Trizol reagent (Invitrogen, Carlsbad, CA, USA). The quantity of RNA was determined by Nanodrop 2000 Spectrophotometer at 260/280 nm. RT-qPCR was performed on the ABI Prism 7900HT Sequence Detection System (Applied Biosystems, Foster City, California, USA) using Power SYBR Green. Primers for the reaction were as follows: Pcna, Forward primer: 5′-TCAGGTACCTCAGAGCAAACG-3′, Reverse primer: 5′-AAGTGGAGAGCTTGGCAATG-3′; Foxg1, Forward primer: 5′-TGGCAAGGCATGTAGCAAA-3′, Reverse primer: 5′-TCCACAGAACGCACCCAC-3′; Ptch1, Forward primer: 5′-AATTCTCGACTCACTCGTCCA-3′, Reverse primer: 5′-CTCCTCATATTTGGGGCCTT-3′; Bax, Forward primer: 5′-GATCAGCTCGGGCACTTTAG-3′, Reverse primer: 5′-TTGCTGATGGCAACTTCAAC-3′; Casp8, Forward primer: 5′-TGCCCAGTTCTTCAGCAATA-3′, Reverse primer: 5′-GCAGGTACTCGGCCACAG-3′; Casp9, Forward primer: 5′-GGCGGAGCTCATGATGTCTGTG-3′, Reverse primer: 5′-TTCCGGTGTGCCATCTCCATCA-3′; GAPDH, Forward primer: 5′-TTGATGGCAACAATCTCCAC-3′, Reverse primer: 5′-CGTCCCGTAGACAAAATGGT-3′.

### Immunohistochemical assay of PH3 and caspase-3

Three control and three NTD embryos on gestation day 11.5 formalin-fixed embryos were paraffin-embedded and cut into 5 μm sections. The sections were dried on a slide dryer at 58 °C for 1 h, then dewaxed and rehydraed by a series of xylol and ethanol rinses using the Leica Autostainer XL (Leica Biosystems Nussloch GmbH, Germany). The Dako antigen retrieval solution was used for heat-activated antigen retrieval (pH 6.0) (Dako, Glostrup, Denmark). The phosphohistone H3 (PH3) (Ser10) antibody (Cell Signaling, 1:400) and the cleaved caspase-3 (Asp175) antibody (Cell Signaling, Boston, MA, USA; 1: 250) were uesd to detect the proliferation and apoptosis of neuroepithelial cells in the sections. Six equal-sized fields were randomly selected and the mean number of positive cells was counted under light microscope.

### Western blotting analysis for PH3 and caspase-3

The appropriate amount of CelLytic MT reagents (Sigma-Aldrich, St. Louis, MO, USA) (20 ml of reagent for 1 g of tissue) was added to embryonic brain tissues (3–4 embryonic brain tissues as one samples) respectively for protein extraction. The samples were then transferred (with lysis/extraction reagent) to a pre-chilled microhomogenizer and centrifuged at 12,000 g for 10 min at 4 °C. The supernatant was isolated and maintained at −80 °C. Protein levels were determined by Nanodrop 2000 Spectrophotometer at 280 nm (Thermo Scientific, Waltham, MA, USA). Proteins were separated by polyacrylamide gel electrophoresis and transferred to polyvinylidene fluoride membranes. After the SDS/PAGE, samples were placed in 5 % skim milk at room temperature for 1 h. The phosphohistone H3 (PH3) (Ser10) antibody (Cell Signaling, 1: 1000) and the cleaved Caspase-3 (Asp175) antibody (Cell Signaling, Boston, MA, USA; 1: 500) were added to the membranes overnight at 4 °C. After that, anti-rabbit secondary antibodies (1: 1000) were added at room temperature for another 2 h. Detection was performed using ECL reagent. Results were analyzed with a gel image processing system.

### Statistical analysis

Data were analyzed using SPSS16.0 software. GARFT activity was analyzed using one-factor analysis of variance (ANOVA), and the comparison between the control and NTDs samples were examined by Student’s *t*-test. All of the data were expressed as mean ± standard deviation (x ± s). *P* < 0.05 was considered as statistically significant.

## Results

### Murine NTDs induced by DDATHF

Embryos were isolated and observed under a dissecting microscope on gestational day 11.5. Results showed that with the increase of DDATHF doses, the rate of embryonic resorption and growth retardation increased. At the dose of 60 mg/kg b/w DDATHF, all of the embryos were resorbed. Meanwhile, the incidence of NTDs varies by the treatment of different DDATHF doses. An ideal animal model should have higher malformation rates and lower mortality rate. At the dose of 40 mg kg/b/w, embryos with NTDs showed the highest rate (30 %) with a lower embryonic resorption rate (37.5 %). Based on this, 40 mg kg-1 b/w was selected as the optimal dose to establish NTDs model in the present study (Table 1).

**Table 1:** Embryonic phenotypes of mice treated by lometrexol (DDATHF)

DDTHF (mg kg^−1^)	Litters (n)	Embryos (n)	Normal n (%)	Resorption n (%)	Growth retardation n (%)	NTDs n (%)	Other malformations n (%)
0	8	62	60 (96.8)	2 (3.2)	0 (0)	0 (0)	0 (0)
15	7	50	47 (94)	2 (4)	1 (2)	0 (0)	0 (0)
30	5	38	29 (76.3)	5 (13.2)	2 (5.3)	1 (2.6)	1^a^ (2.6)
35	4	31	15 (48.4)	8 (25.8)	4 (12.9)	2 (6.5)	1^a^;1^b^ (6.5)
40	9	65	8 (12.3)	21 (32.3)	14 (21.5)	20 (30.8)	2^a^ (3.1)
45	5	34	2 (5.9)	21 (61.8)	9 (26.5)	2 (5.9)	0 (0)
60	4	29	0 (0)	29 (100)	0 (0)	0 (0)	0 (0)

*NTDs* neural tube defects

^a^craniofacial malformation

^b^polydactyly

### Morphological and pathological changes of embryos after DDATHF intervention

**Fig. 1:**
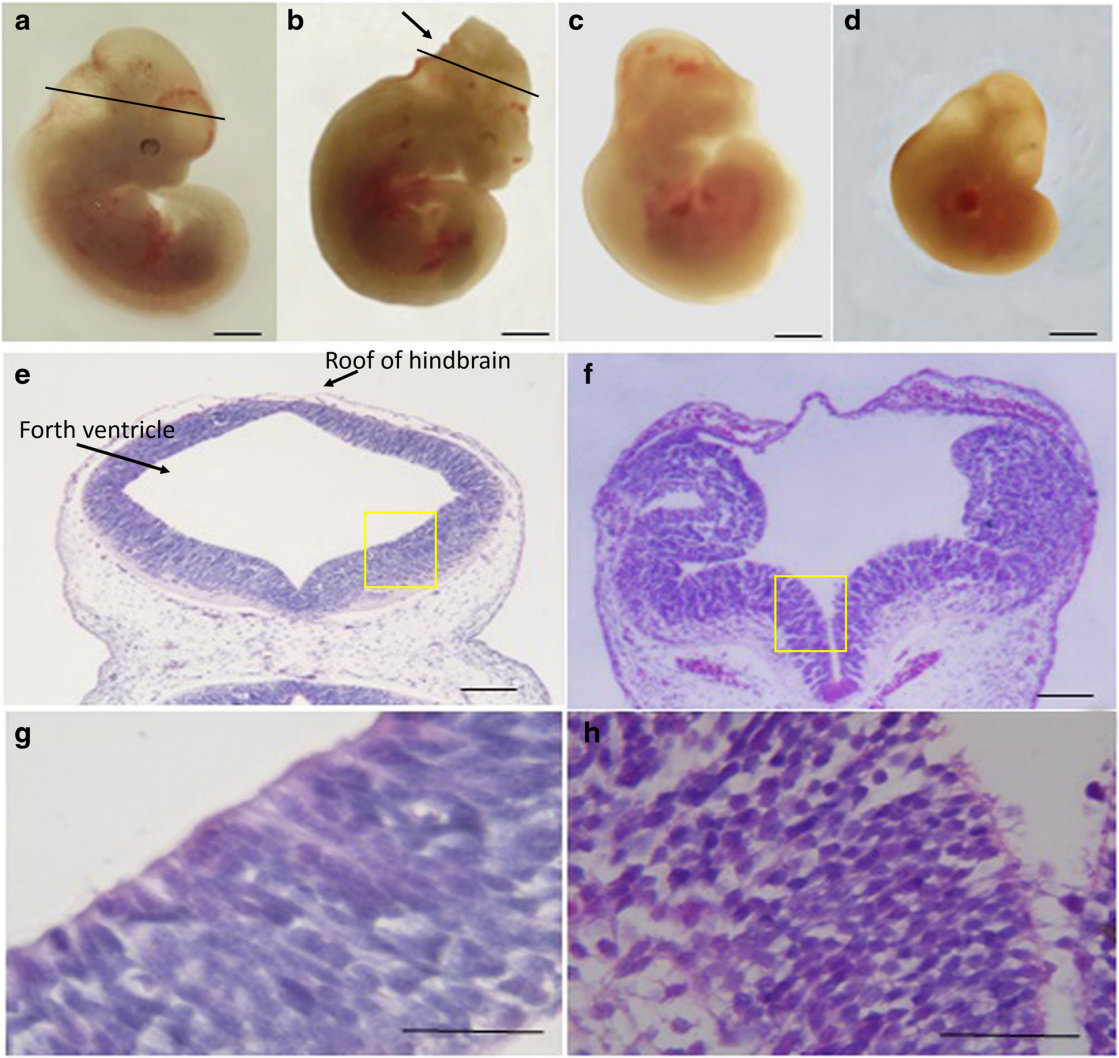
Morphological changes of normal and abnormal embryos on gestational day 11.5 (**a**–**d**). Observation of embryonic development under a dissecting microscope (Scale bars: 1 mm). **a** Normal embryos (from control group); **b**–**d** abnormal embryos from the lometrexol 40 mg/kg body weight group, the NTD embryos showed failure closure of hindbrain of the neural tube (**b**, *arrow*), craniofacial malformation (**c**) and growth retardation (**d**) separately. **e**–**h** Photomicrographs of coronal sections at the same plate of hindbrain (black lines in **a** and **b**) using hematoxylin and eosin staining (Scale bars: **e**, **f** 100 μm; **g**, **h** 50 μm). (**e**, **g**) Normal embryos; (**f**, **h**) NTD embryos. **g** was magnifying (**e**). **h** was magnifying (**f**). exencephalycraniofacial malformation

Neural tube defects (NTDs) are congenital malformations due to the failure of neural tube closure during embryonic development, including anencephaly, spina bifida and encephalocele. In this study, we observed the difference of the phenotypes between the normal and NTD embryo on gestational day 11.5 via stereoscopic microscope. Then we investigated the tissue structure characteritic of the normal and NTD brain by the methods of H&E staining. From the level of anatomy, the control embryo showed full appearance, normal formation of forebrain, midbrain and afterbrain, smooth and integrated surface of rachis, visible otic vesicle and optic vesicle, curled and long thin tail (Fig. 1a, Additional file 1: Figure S1). NTDs embryos showed exencephaly. Although forebrain, midbrain and hindbrain have been formed, the margin were obscure. However, the surface of rachis were smooth (Fig. 1b, Additional file 1: Figure S1). H&E staining of normal embryos showed neural tube full closure, lumen regular, neuroepithelial cells arranged in neat rows (Fig. 1e, g). H&E staining of NTDs embryos showed unfused neural tube hindbrain apical plate which were wrapped by a layer of amniotic membrane and stroma, irregular arranged neuroepithelial cells (Fig. 1f, h). Besides, other malformations including craniofacial abnormalities (Fig. 1c) and growth retardations (Fig. 1d) were also observed.

### GARFT activity decreased after DDATHF injection

**Fig. 2:**
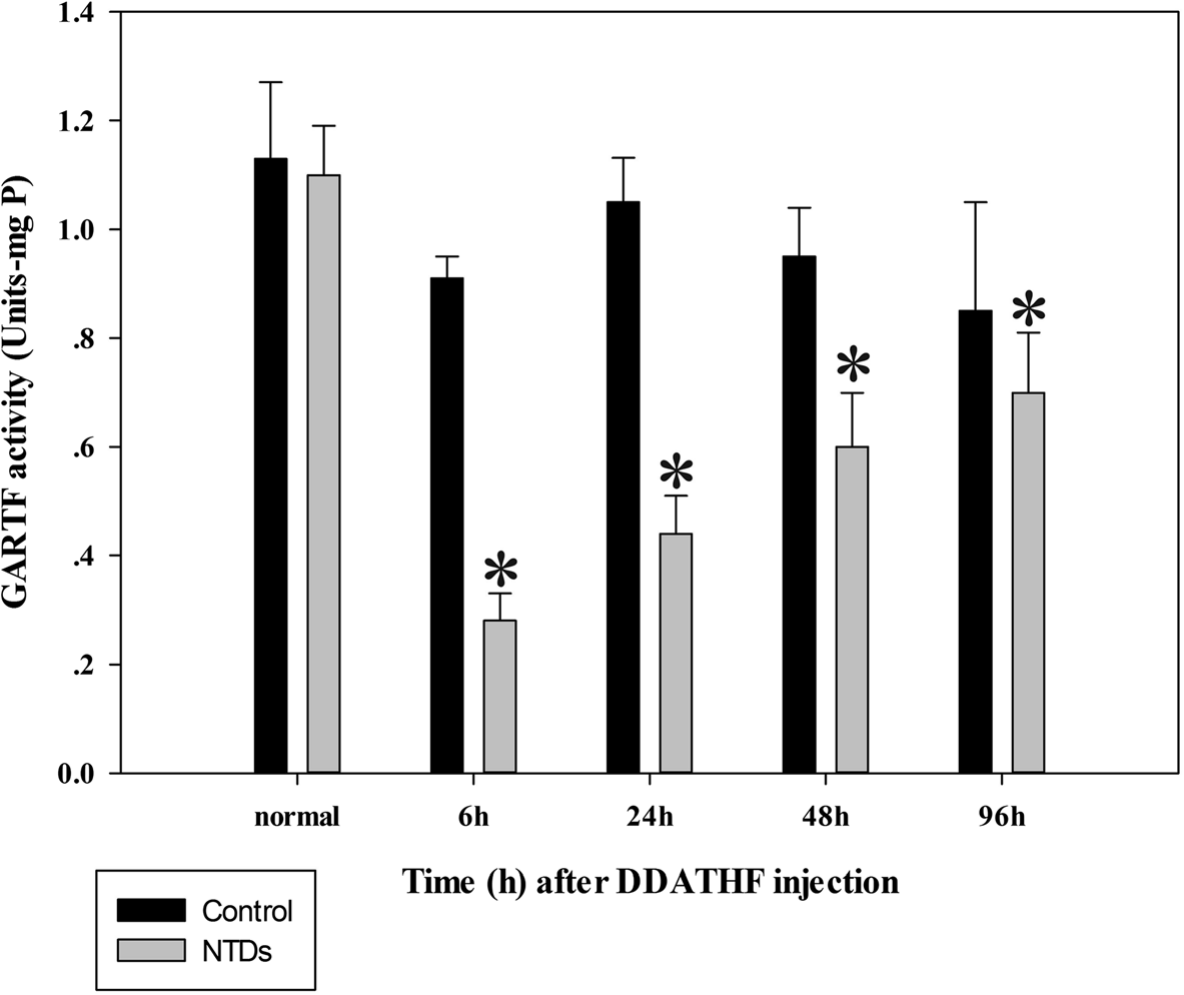
Glycinamide ribonucleotide formyl transferase (GARFT) activities of embryonic brain tissues. DDATHF were intraperitoneally (I.P.) injected to the pregnant mice at the optimal dose, 40 mg kg^−1^ body weight (b/w) on the gestation day 7.5. Embryonic brain tissue samples (3–4 embryonic brain tissues as one sample) were collected according to the noted time. The GARTF activities were determined as described in Methods. Data were expressed as means ± SD (*n* = 6). *P < 0.05, vs. GARFT activities of normal embryonic tissues

GARFT activity in embryonic tissue of control and DDATHF treated was detected at 0, 6, 24, 48 and 96 h after i.p. injection of optimal DDATHF dose on gestational day 7.5 to confirm the effects of DDATHF on de novo purine synthesis. Compared with the control embryo, GARFT activity was maximally inhibited after at 6 h after DDATHF injection, and thereafter gradually increased with time but remained significantly lower than control even at 96 h. (Fig. 2a). The result showed that GARFT activity was inhibited by DDATHF.

### Changes of ATP, GTP, dATP and dGTP levels after DDATHF injection

**Fig. 3:**
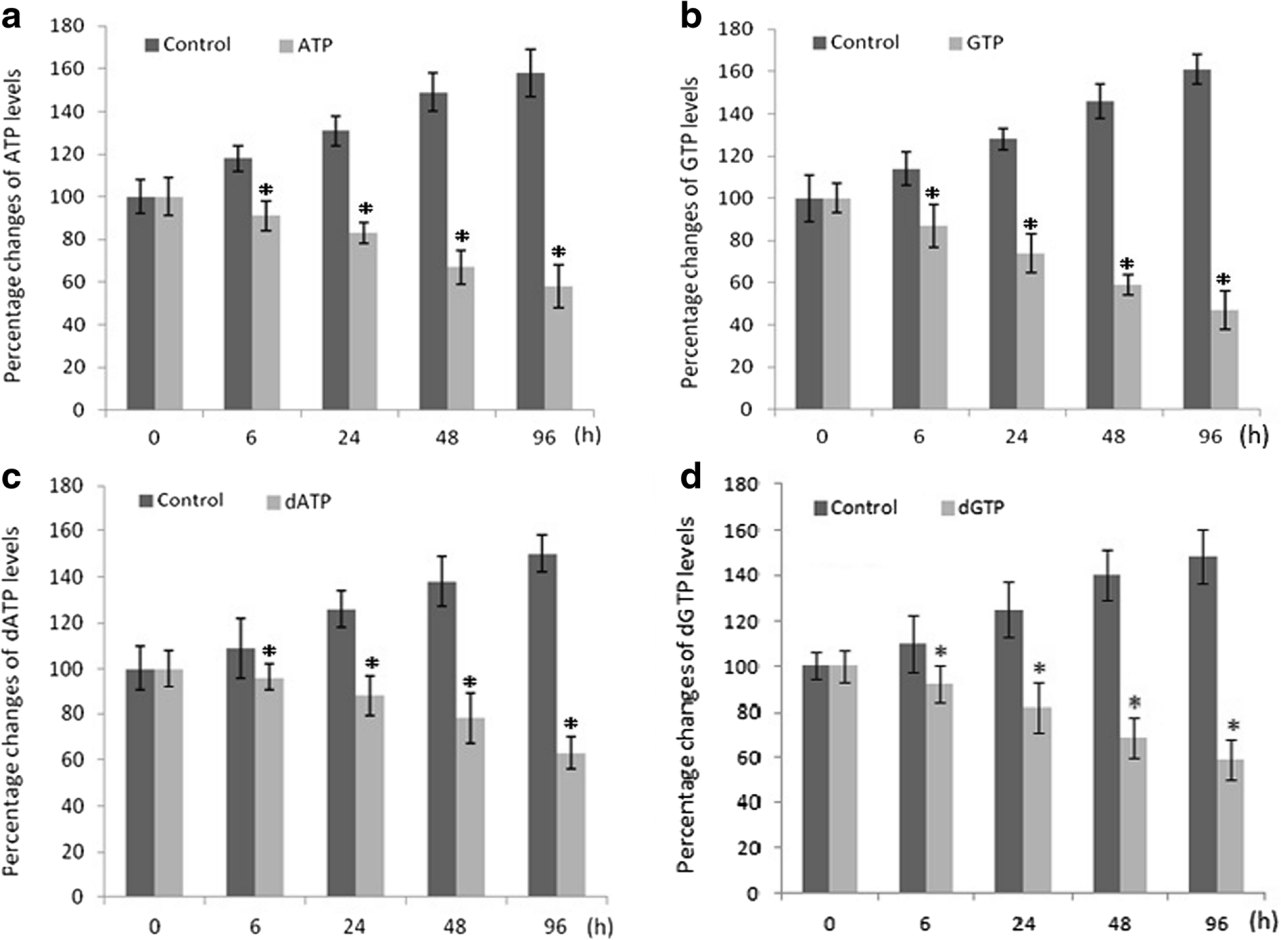
Histograms of time-dependent alterations of ATP, GTP, dATP and dGTP levels in embryonic brain tissue on gestation day 11.5. At the indicated time after dosing (40 mg/kg body weight DDATHF), the samples (3–4 embryonic brain tissues as one sample) were collected and handled as described in Methods. The black bars show are the control groups, the grey bars show the ATP, GTP, dATP, dGTP levels respectively of the DDATHF injected mice *P < 0.05

ATP, GTP, dATP and dGTP levels were measured using high performance liquid chromatography following DDATHF treatment at 0, 6, 24, 48 and 96 h, respectively. Compared to control, levels of ATP, GTP, dATP and dGTP of NTDs embryonic brain tissue decreased significantly at 6 h after DDATHF treatment, and more significantly over time (Fig. 3). The changes of ATP and GTP were more pronounced than dGTP and dATP.

### Analysis of proliferation and apoptosis by RT-qPCR

**Fig. 4:**
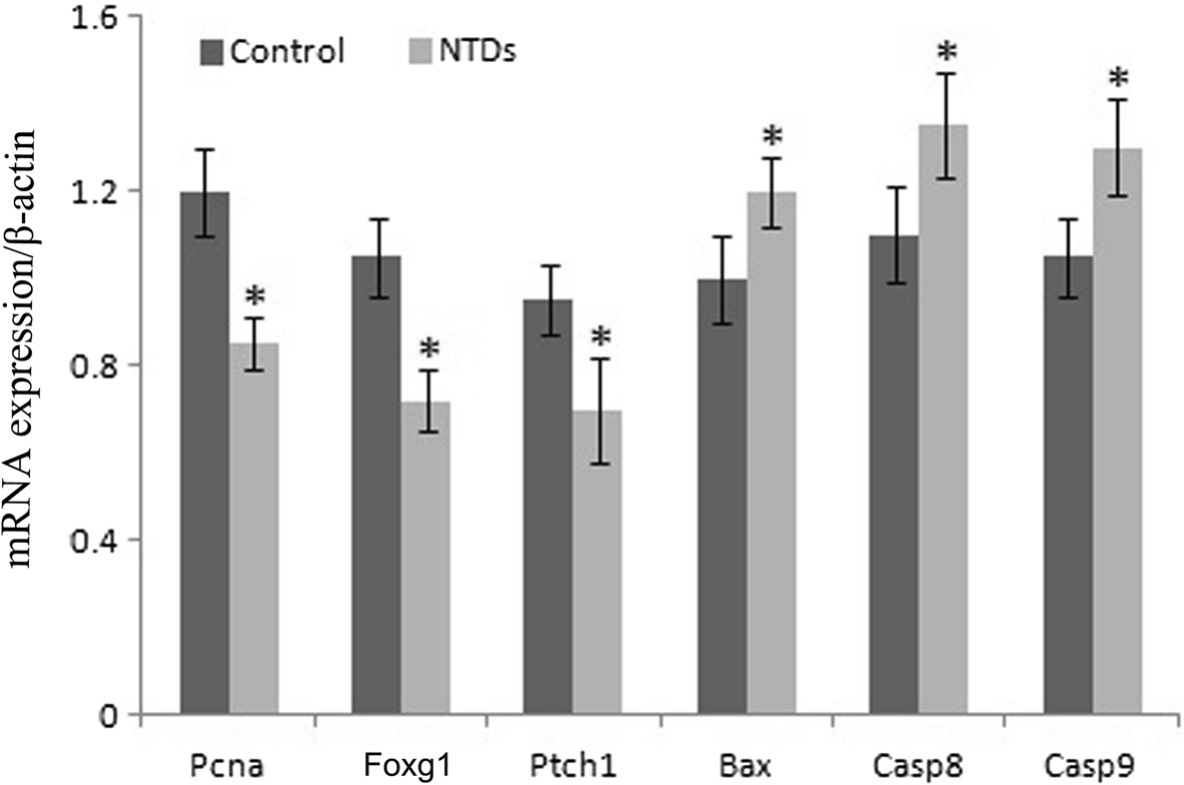
The mRNA expression of proliferation and apoptosis genes were measured by use of reverse transcription-quantitative polymerase chain reaction. DDATHF were intraperitoneally (I.P.) injected to the pregnant mice at the optimal dose, 40 mg kg-1 body weight (b/w) on the gestation day 7.5. Embryonic brain tissue samples were collected on the gestation day 11.5 *P < 0.05 NTDs versus control. NTDs, neural tube defects

Samples of control (3–4 normal embryonic brain tissues per litter as one sample) and NTDs group (3–4 NTD embryonic brain tissues per litter as one sample) had three biological replicates, respectively. Proliferation-related genes (Pcna, Foxg1 and Ptch1) and apoptosis-related genes (Bax, Casp8 and Casp9) were measured to investigate the proliferation and apoptosis in NTDs by RT-qPCR (Fig. 4). mRNA gel picture was provided as Additional file 2: Figure S2. Results showed that compared to control group, expression of proliferation-related genes were significantly decreased and expression of apoptosis-related genes were significantly increased in NTD groups.

### Analysis of proliferation in neuroepithelial cells

**Fig. 5:**
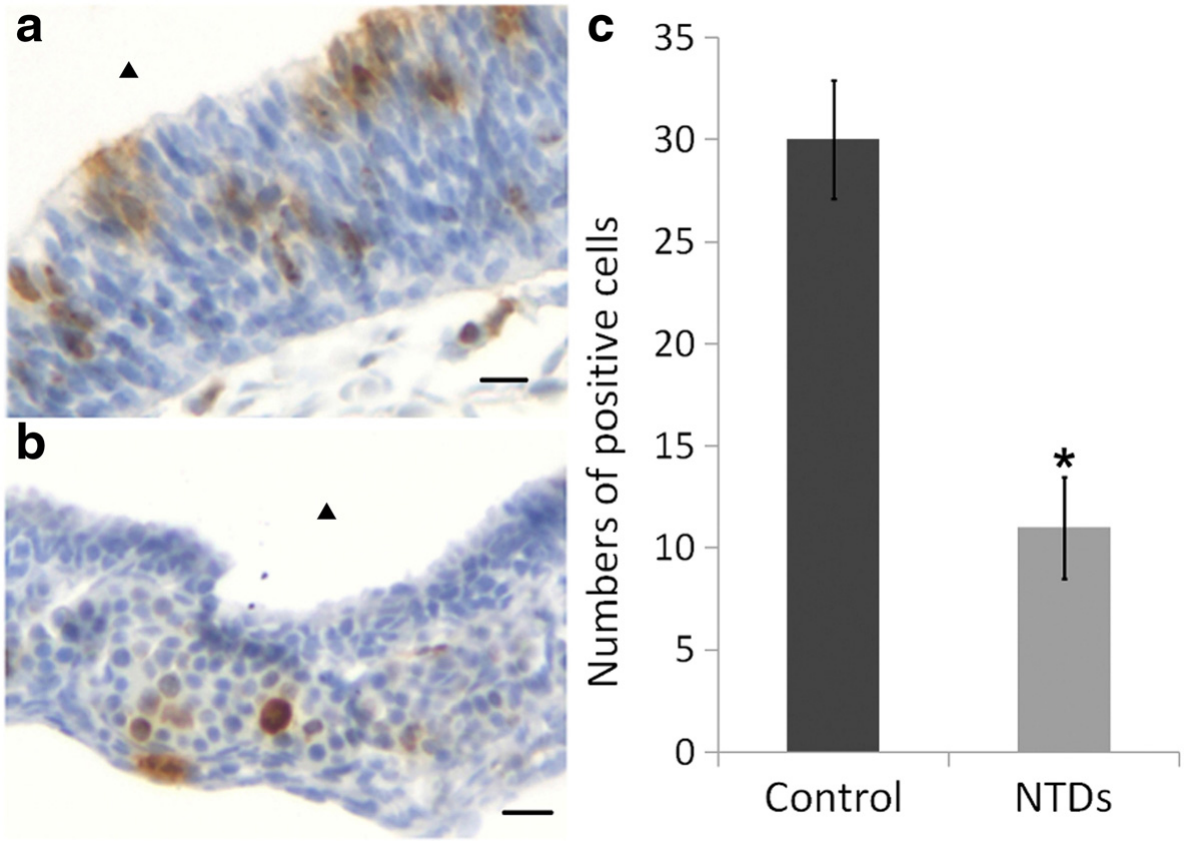
Immunostaining of PH3 in neuroepithelium from control and NTDs embryos on gestation day 11.5. **a** Immunostaining of PH3 in neuroepithelium of hindbrain from control embryo; **b** immunostaining of PH3 neuroepithelium of hindbrain from NTD embryo; **c** quantification of the number of PH3 positive cells. Bar = 100 μm. ▲ nfourth ventricle, *P < 0.05 NTDs versus control. NTDs, neural tube defects

To further verify the results of RT-qPCR, we performed PH3 staining to assess the proliferation of neuroepithelial cells by the use of immunohistochemical assay, which showed that PH3 staining in NTD embryos (Fig. 5b) was less obvious than that in control embryos (Fig. 5a) (*P* < 0.05). Furthermore, the protein levels of PH3 in embryonic brain tissue were analyzed by western blot (Fig. 7a), and the results showed that the expression of PH3 was significantly reduced.

### Analysis of apoptosis in neuroepithelial cells

**Fig. 6:**
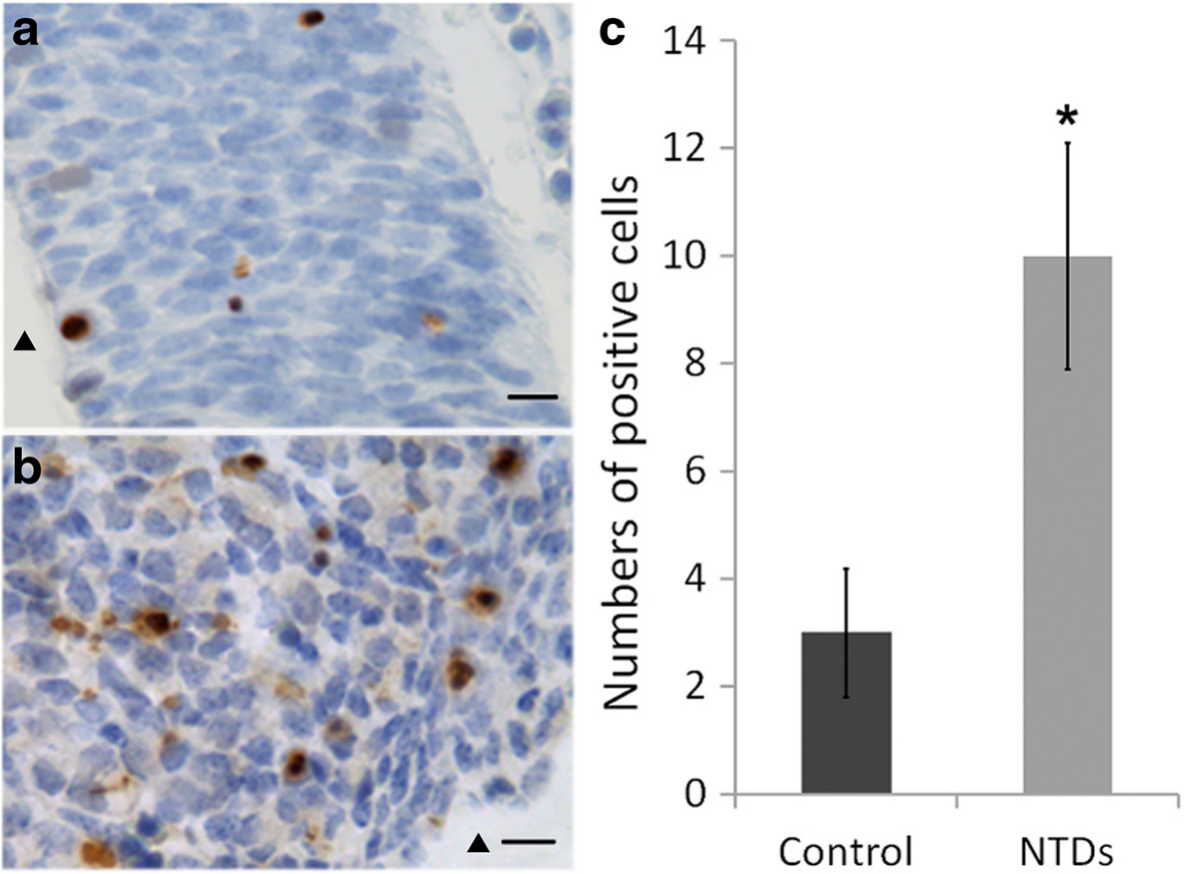
Immunostaining of activated caspase-3 in neuroepithelium from control and NTDs embryos on gestation day 11.5. **a** Immunostaining of activated caspase-3 in neuroepithelium of hindbrain from control embryo; **b** immunostaining of activated caspase-3 in neuroepithelium of hindbrain from NTD embryo; **c** quantification of the number of activated caspase-3 positive cells. Bar = 50 μm. ▲ fourth ventricle. *P < 0.05 NTDs versus control. NTDs, neural tube defects

**Fig. 7:**
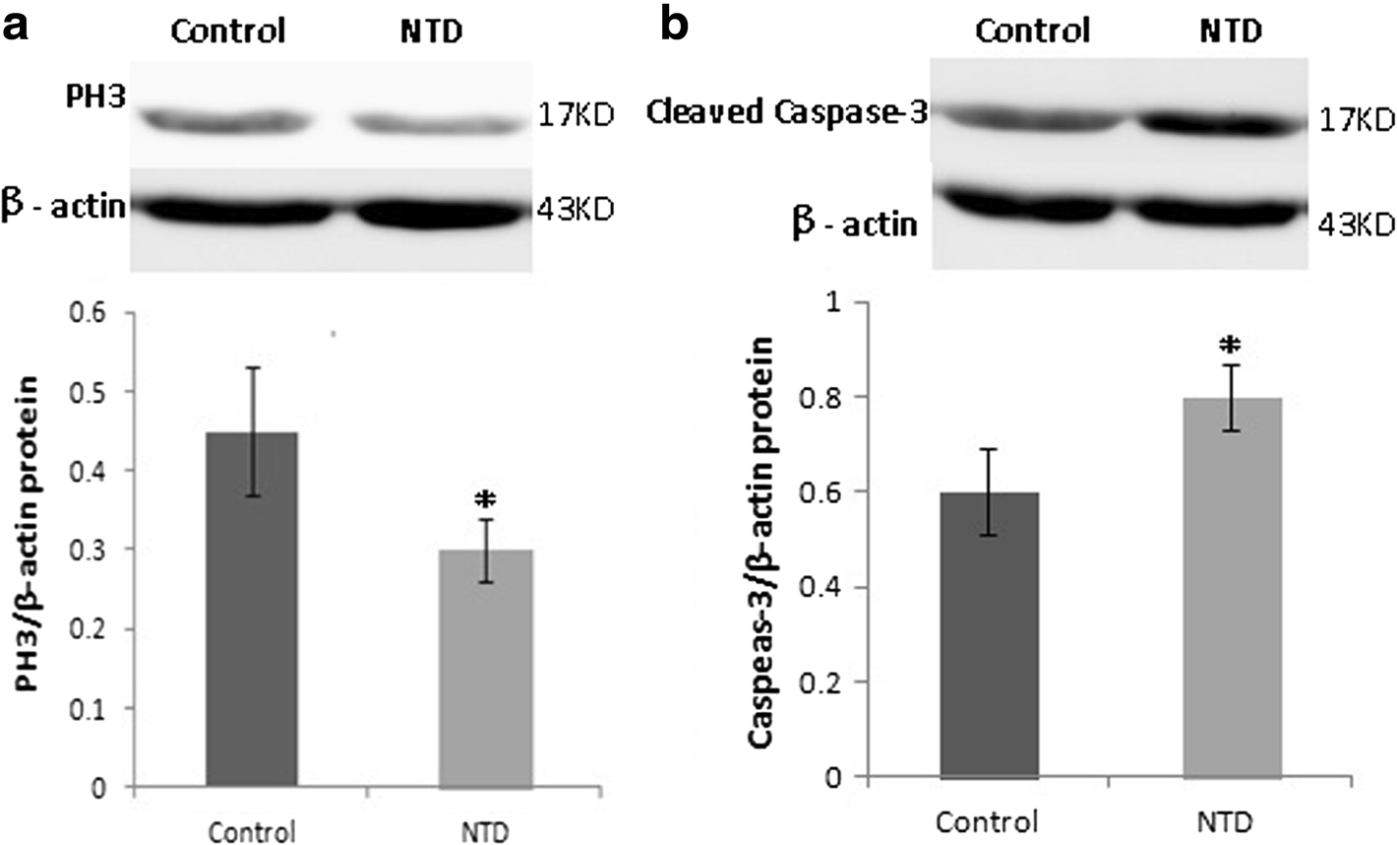
Western blot analysis of PH3 (**a**) and activated caspase-3 (**b**) in control and embryos with NTDs in embryonic brain tissue on gestation day 11.5. NTDs, neural tube defects. Date were expressed as meas ± SD (*n* = 6)

To investigate apoptosis in neuroepithelial cells, Caspase-3 staining was performed using immunohistochemical assay. The results revealed that staining of Caspase-3 in NTD embryos (Fig. 6b) was more obvious than that in the control embryos (Fig. 6a) (*P* < 0.05). We also determined the expression levels of Caspase-3 protein in embryonic brain tissue using western blot. The results showed that the expression of cleaved caspase-3 protein were significantly increased in embryonic brain tissue of NTDs (Fig. 7b), which were consistent with the RT-qPCR results. These results indicate that abnormal proliferation and apoptosis exist in NTDs induced by DDATHF.

## Discussion

In the present study, NTDs were successfully induced by disturbing purine metabolism via DDATHF. Pregnant mice were intraperitoneally injected with DDATHF on gestational day 7.5 and embryos were observed on gestational day 11.5. The group treated with 40 mg kg^−1^ DDATHF caused the highest incidence of NTDs (30.8 %) with lowest lethality. Therefore, it was selected as the optimal dose (40 mg/kg body weight) to establish murine NTDs. As gestational day 7.5 is the critical period for neural tube closure [18–20], lack of closure in the hindbrain was primarily observed in embryos with NTDs. According to the opinion in Copp et al. [21], when closure one fails, almost the entire neural tube from the midbrain to the lower spine remains open, which is a condition known as craniorachischisis. Embryos in which closure two fails or is disrupted at the anterior or midbrain–hindbrain neuropores, have exencephaly. The specific failure of closure three leads to anencephaly that is confined to the forebrain region, often in association with a split-face malformation. In our results of exencephaly, closure two or three was mainly affected. Craniofacial malformation was caused by closure three failure. Besides, DDATHF also caused growth retardation. These results indicate that DDATHF induced congenital malformations, especially NTDs. The study has investigated the effects of purine dysmetabolism on the development of NTDs and we have successfully established murine NTDs with a high incidence of NTDs, which is in accordance with our aims.

DDATHF is a specific and classical inhibitor against GARFT. GARFT is a key enzyme in the de novo synthesis of purines without any inhibitory effects on enzymes involved in folate synthesis and conversion [9, 22]. Polyglutamated form of DDATHF binds with GARFT 100 times more potently than DDATHF [23]. In the present study, GARFT activity in embryonic brain tissue was obviously decreased by DDATHF treatment and remained significantly lower than control even at 96 h following DDATHF administration. Levels of ATP, GTP, dATP and dGTP in embryonic brain tissue were also significantly reduced by DDATHF treatment. Purines are mainly used for the biosynthesis of nucleic acids. Our results demonstrate that DDATHF impaired the purine metabolism by inhibition of GARFT, leading to the development of embryonic malformations especially NTDs. The study has provided direct evidence for the association between purine dysmetabolism and NTDs for the first time. It may be one of the mechanisms in folate deficient NTDs.

Impairment of purine biosynthesis via DDATHF may affect neural tube closure by influencing cell apoptosis and proliferation. During neural tube closure, cell differentiation, proliferation, apoptosis and other biological processes are necessary [24, 25]. Cell proliferation and apoptosis need to maintain dynamic equilibrium during the development of neural tube. If the equilibrium was broken, NTDs would occur. Purine dysmetabolism may affect DNA, RNA synthesis, cell proliferation and apoptosis. Recently, Xia Cong MM et al. [26] found that increased expression of GARFT was associated with promoted cell proliferation in liver cancer. However, cell proliferation was inhibited as GARTF was depleted evidenced by decreased expression of proliferating cell nuclear antigen. Anthony Ng et al. found that, Zygotic gart and paics mutants lead to purine dysmetabolism, ATP and GTP depletion and disturbed DNA synthesis during S phase in the zebrafish embryos. Besides, decreased proliferation and more apoptosis were also found. These abnormalities ultimately result in severe developmental defects [27]. Studies have indicated that DDATHF induced cell cycle arrest in CEM and HL60 cells [28]. Our previous study have found that Foxg1 and Ptch1 which are candidate genes in NTDs were closely related to the development of NTDs. We would like to identify whether Foxg1 and Ptch1 express the same as our previous study. In the study, mRNA levels of the proliferation-related genes (Pcna, Foxg1 and Ptch1) were significantly decreased. Immunohistochemical assay and Western blot results showed that the protein levels of PH3, a mitosis marker for cell proliferation [29, 30], was also inhibited. These results suggest that cell proliferation was disturbed in NTDs induced by purine dysmetabolism. Meanwhile, increased expression of apoptosis related genes (Bax, Casp8 and Casp9) and cleaved Caspase-3 in NTD embryonic brain tissue indicates excess apoptosis in NTDs induced by DDATHF. Furthermore, cells with positive immunohistochemical stain of Caspase-3 increased in neuroepithelial cells. These results implied that DDATHF may cause NTDs through impairing the de novo synthesis of purines and imbalance between proliferation and apoptosis was involved in the development of NTDs.

## Conclusions

DDATHF induced the serve birth defects in mice, especially NTDs. We have successfully established an animal model of NTDs induced by purine dysmetabolism in the present study. Abnormal cell proliferation and apoptosis were found in the model, which was consistent with previous studies. Therefore, folate may act through purine metabolism to affect neural tube development.

## Additional files

Additional file 1: Figure S1. The development of control and DDATHF-treated (40 mg/kg body weight) embryos on gestation day 11.5 observed under a dissecting microscope. (A, B) the control embryos on gestational day11.5; (C, D) embryos with NTDs .(B, D) The spine of control and NTD embryos are closed well. (magnification 10×). (TIF 61 kb) Additional file 2: Figure S2. mRNA gel picture. (TIF 878 kb)

## Abbreviations

NTDs: Neural tube defects
GARFT: Glycinamide ribonucleotide formyl transferase
DDATHF: Dideazatetrahydrofolate
DHFR: Dihydrofolate reductase
THF: Tetrahydrofuran
GAR: Glycinamide ribonucleotide
GAR: Glycinamide ribonucleotide
FGAR: Glycinamide ribonucleotide formyl-acid
MTX: Methotrexate
H&E: Hematoxylin and eosin
HPLC: High performance liquid chromatography
TBAH: Tetrabutyl ammonium hydroxide
PH3: Phosphohistone H3
ANOVA: One-factor analysis of variance
