# Supplementary figures and images for: The effect of inhibiting glycinamide ribonucleotide formyl transferase on the development of neural tube in mice
Source: Nutr Metab (Lond). 2016 Aug 23;13(1):56. doi: 10.1186/s12986-016-0114-x (PMC4994272; doi:10.1186/s12986-016-0114-x)

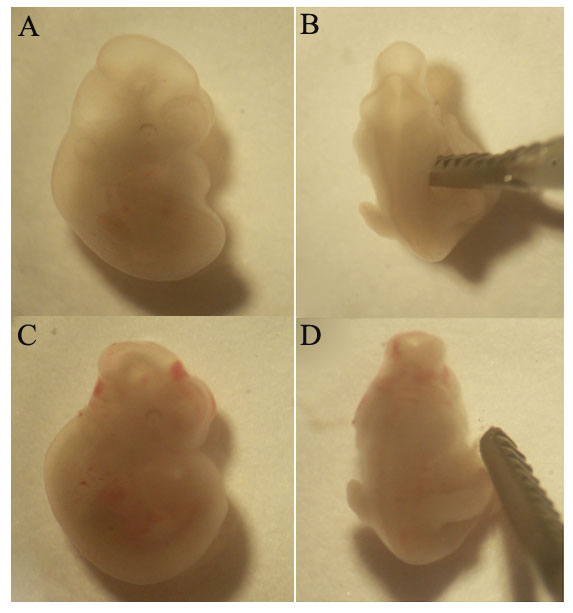

Supplement: Additional file 1: Figure S1. — The development of control and DDATHF-treated (40 mg/kg body weight) embryos on gestation day 11.5 observed under a dissecting microscope. (A, B) the control embryos on gestational day11.5; (C, D) embryos with NTDs .(B, D) The spine of control and NTD embryos are closed well. (magnification 10×). (TIF 61 kb) [file 12986_2016_114_MOESM1_ESM.tif]

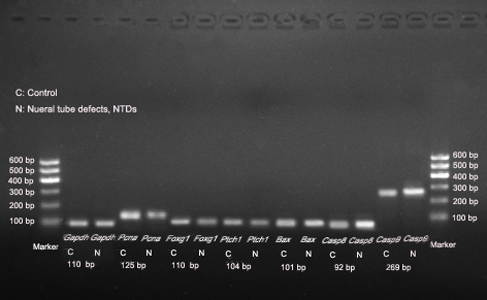

Supplement: Additional file 2: Figure S2. — mRNA gel picture. (TIF 878 kb) [file 12986_2016_114_MOESM2_ESM.tif]
